# Global coordination in adaptation to gene rewiring

**DOI:** 10.1093/nar/gku1366

**Published:** 2015-01-06

**Authors:** Yoshie Murakami, Yuki Matsumoto, Saburo Tsuru, Bei-Wen Ying, Tetsuya Yomo

**Affiliations:** 1 Graduate School of Information Science and Technology, Osaka University, 1-5 Yamadaoka, Suita, Osaka 565-0871, Japan; 2 Graduate School of Life and Environmental Sciences, University of Tsukuba, Tsukuba, Ibaraki 305-8572, Japan; 3 Graduate School of Frontier Biosciences, Osaka University, Suita, Osaka 565-0871, Japan; 4 Exploratory Research for Advanced Technology (ERATO), Japan Science and Technology Agency (JST), Suita, Osaka 565-0871, Japan; 5 Earth-Life Science Institute, Tokyo Institute of Technology, Meguro, Tokyo 152-8550, Japan

## Abstract

Gene rewiring is a common evolutionary phenomenon in nature that may lead to extinction for living organisms. Recent studies on synthetic biology demonstrate that cells can survive genetic rewiring. This survival (adaptation) is often linked to the stochastic expression of rewired genes with random transcriptional changes. However, the probability of adaptation and the underlying common principles are not clear. We performed a systematic survey of an assortment of gene-rewired *Escherichia coli* strains to address these questions. Three different cell fates, designated good survivors, poor survivors and failures, were observed when the strains starved. Large fluctuations in the expression of the rewired gene were commonly observed with increasing cell size, but these changes were insufficient for adaptation. Cooperative reorganizations in the corresponding operon and genome-wide gene expression largely contributed to the final success. Transcriptome reorganizations that generally showed high-dimensional dynamic changes were restricted within a one-dimensional trajectory for adaptation to gene rewiring, indicating a general path directed toward cellular plasticity for a successful cell fate. This finding of global coordination supports a mechanism of stochastic adaptation and provides novel insights into the design and application of complex genetic or metabolic networks.

## INTRODUCTION

Gene rewiring, or an alteration of preexisting genetic connections, is a common evolutionary phenomenon (1–3). In nature, rewiring is caused by mutations in promoters or by the transposition of genomic sequences to other loci and is considered a means of conferring novel gene regulation and/or essentialities in evolution (4–7). Such genetic alterations may lead to either survival or extinction for the organism, as the alterations could in turn lead to disturbances in existing regulatory patterns. Nevertheless, increasing experimental studies indicate that cells can survive genetic rewiring with a high probability. For example, newly connected gene networks have been shown to be tolerated by bacteria (8), and successful adaptation has been observed in bacterial and yeast cells undergoing gene rewiring in response to external changes (9–11).

Successful cell fates were largely explained by stochastic switching of rewired genes (10,12–14). That is, the stochasticity of gene expression (15–19) occasionally produces fit cells in response to unforeseen environments (i.e. stochastic adaptation, Supplementary Figure S1A) (10,13,20–21). Stochastic adaptation does not require specific regulation, but it largely relies on cell diversity within a genetically identical population (22,23). Therefore, stochastic adaptation is considered a universal survival strategy in living cells (20,24). Despite previous experimental results and a proposed mechanism for adaptation, how often the stochastic expression of rewired genes results in adaptation and whether stochasticity in rewired genes is solely sufficient for a successful cell fate are not known.

Current studies identified common features of global changes in living organisms, which prompted us to consider a general mechanism for adaptation *via* transcriptome reorganization under cellular plasticity in rewired cells. Pioneering transcriptome studies commonly observed random patterns in global gene expression, which indicated that cellular plasticity contributed to the adaptation of rewired cells (11,25). In comparison, common patterns of transcriptome reorganization in response to external stress were largely reported in native cells (i.e. wild-type strains). For example, the correlation between growth rate and gene expression was universal regardless of variations in external perturbations in yeast and bacteria (26–29). Recent studies on cellular networks illustrated the general mechanisms of network dose compensation (30), metabolic restriction (31) and the coordination between proteome and metabolic signals (32). In comparison, whether and how the global reorganization of gene expression contributes to stochastic adaptation is not clear.

Previous studies using several genetic structures (9–11) were insufficient to reach a general conclusion of the probability of stochastic adaptation or any common patterns in transcriptome reorganization. Therefore, systematic surveys of rewiring cells with varied cell fates were required to determine common properties and investigate organizational principles of living systems (33). This study performed a systematic survey of gene rewiring under a defined stress condition to identify inherent survival approaches of cells and provide novel insights into cellular plasticity. Genetic disruptions were introduced by rewiring the structural genes within the *His* operon (34–36) to a monostable synthetic gene circuit at other chromosomal sites in *Escherichia coli* (37). Because the rewired gene essential for histidine biosynthesis was controlled by a foreign promoter, the stochastic switching of the rewired gene could provide the opportunity for survival from histidine starvation (10). This study first reported three different cell fates that were mediated by stochastic switching of gene expression, which were designated good survivors, poor survivors, and failures. The stochasticity of the rewired genes and reorganization in global gene expression were evaluated to identify similar and distinct features that corresponded to final cell fates. In conclusion, the stochasticity of the rewired gene itself was insufficient to reach a successful cell fate, but the coordinated reorganization of global gene expression within a restrictive dimension was essential. These results indicate a certain level of universal discipline during stochastic adaptation, which is represented by cellular plasticity in rewired cells.

## MATERIALS AND METHODS

### Strains

**Figure 1.**
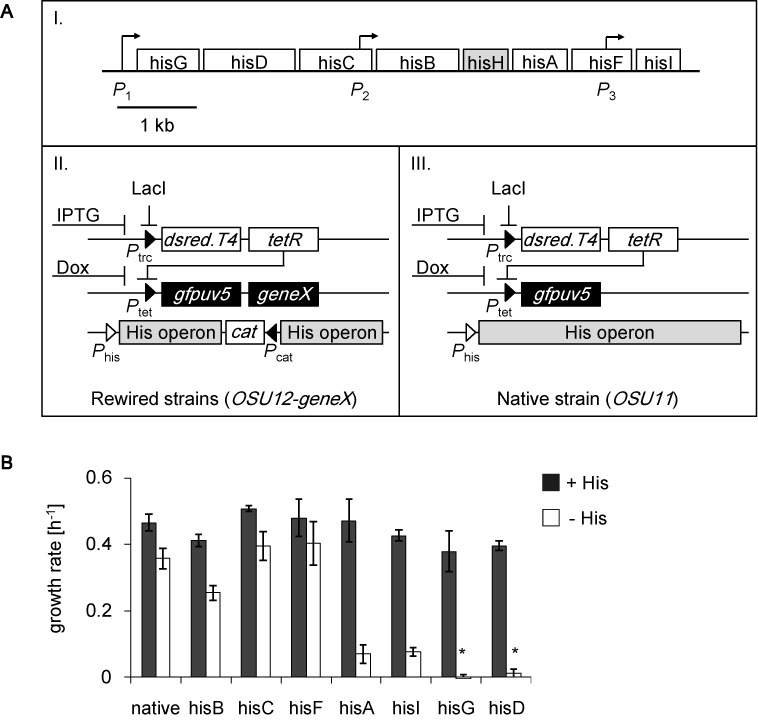
Cell fates of gene rewiring. (**A**) Schematic diagram of the rewired strains. Boxes I, II and III represent the structural genes of the *His* operon, and the genetic structures of rewired strains and the native strain, respectively. Except for *hisH* (gray, which function with *hisF*), all other structural genes were subjected to reconstruction. Both the native strain (*OSU11*) and the rewired strains (*OSU12-geneX*) carried the monostable synthetic gene circuit including the *rfp* (*dsred.T4*) and *gfp* (*gfpuv5*) reporter genes. *OSU11* retains the native *His* operon at its native chromosomal locus. Members of the *OSU12-geneX* series each have a deficient *His* operon and a single rewired structural gene (*hisG, hisD, hisC, hisB, hisA, hisF* and *hisI*). (**B**) Growth fitness in the presence and absence of histidine. Exponential growth rates were evaluated in both histidine-rich (+His, filled) and histidine-free (−His, open) media. Asterisks indicate that significant cell growth was undetected within 2 days.

The *OSU11* and *OSU12-geneX E. coli* strains (Figure 1A) were constructed as previously described (10,37). Both strains carry a core genetic circuit, *P_tetA_–gfpuv5* and *P_trc_–dsred.t4–tetR–PEM7–zeo*, located in their genomes at the *galK* and *intC* sites, respectively. *PtetA–gfpuv5* consists of the promoter *PtetA* and the mutated *gfp* gene (*gfpuv5*) encoding green fluorescent protein (GFP), and *P_trc_–dsred.t4–tetR–PEM7–zeo* is comprised of the promoter *P_trc_, dsred.t4* encoding red fluorescent protein (RFP), *tetR* encoding the repressor protein and the independent expression unit of the zeocin resistance gene, *zeo*, with its promoter PEM7. The correlation between the co-expressed *gfp* gene and the downstream rewired genes were previously demonstrated (10,37).

### Cell culture and growth rate

*Escherichia coli* cells were cultured in minimal medium in the presence of 1 mM histidine at 37°C for several passages to reach a constant growth rate, as previously described (10). Single colonies from the resultant populations were isolated and stored at −80°C until analysis. All cell cultures were inoculated using the single colony derived glycerol stocks. Precultures were incubated with 100 μM isopropyl β-d-1-thiogalactopyranoside (IPTG) to induce full expression of the TetR repressor, which was reported by RFP expression. Exponentially growing cells were subsequently transferred to fresh media containing 50 or 100 nM doxycycline hydrochloride (Dox) to induce the expression of the downstream rewired genes, as reported by GFP expression. Following the induction of the rewired genes, the cultures were subjected to both histidine-depleted (–His) and histidine-rich (+His) conditions for additional analysis. Under the conditions of +His and –His (>10 h) conditions, the initial and final cell concentrations were controlled at ∼10^3^ and ∼10^7^ cells/ml, respectively. Under the conditions of –His (10 min) and –His (2 h) conditions, the cell concentrations of the precultures (before transfer) were maintained at ∼10^7^ cells/ml. The cells that were harvested 10 min or 2 h after transfer were counted to confirm their final concentrations, which were often close to their initial concentrations. The growth rate (h^−1^) was evaluated by the speed with which the number of offspring increases during the exponential growth phase, and calculated according to the following commonly used (10,29,38) formula: growth rate (*μ_t_*) = ln(*C_t_*/*C*_0_)/*t*, where *C_t_, C*_0_ and *t* represent the final and initial cell concentrations (cells/ml) and the culture time from initial to final time points (h), respectively.

### Histidine depletion

Aliquots of 350 μl of exponentially growing cells (10^6^–10^7^ cells/ml) were harvested by centrifugation at 8000 rpm for 1 min at 37°C using spin columns (Ultrafree-MC Centrifugal Filter Units, 0.22 μm; Millipore). After discarding the flow-through fraction, the cells were washed with 350 μl of the identical medium without histidine. After an additional centrifugation, the cells were suspended in 350 μl of fresh, histidine-free medium. The concentration of the cell suspension was determined using flow cytometry, and cells were then inoculated at 10^6^–10^7^ cells/ml (slow growth strains) or 10^4^–10^6^ cells/ml (other strains) in 5 mL of the identical histidine-free medium.

### Flow cytometry

The cell concentration, gene expression (fluorescence intensity), and cell volume of *E. coli* cells were determined using a flow cytometer (FACSCanto™ II; Becton Dickinson) equipped with a 488-nm argon laser, a 515–545-nm emission filter (GFP), and a 563–589-nm emission filter (RFP). The following PMT voltage settings were applied: forward scatter (FSC), 450; side scatter (SSC), 400; GFP, 500; and RFP, 600. The flow rate for the sample measurements was set to low. Each calculation was performed using 10 000 collected cells. Cell samples were mixed with fluorescent beads (Fluoresbrite YG Microspheres, Calibration Grade 3.00 μm; Polysciences) to calculate the cell concentration. Daily detection accuracy was monitored using eight-peak beads (SPHERO™ Rainbow Calibration Particles (eight peaks), 3.0–3.4 μm)) for data calibration.

### FCM analysis

The data obtained by flow cytometry were converted to the fcs2.0 format and analyzed by custom-designed scripts written in R (39) using the free packages flowCore and flowViz, which are available from the Bioconductor web site (http://www.bioconductor.org/). Fluorescent beads loaded for the calculation of cell concentration, cell debris and systematic errors resulting from events that occurred at the bottom or top of the instrument's range were eliminated as previously described (10,14). The protein abundance of the rewired genes, which are represented by GFP bias, was calculated by dividing the green fluorescence value (GFP FI) by the red fluorescence value (RFP FI). The relative cell size was evaluated by the FSC value.

### Microscopic observation

Cell samples for FCM analysis were applied to a microscopic observation chamber. A volume of 1 μl of exponentially growing cells in the presence or absence of histidine was placed on a glass slide and covered with a cover slip. For membrane staining, 1 μl of a 200 mg/ml solution of FM 4–64 (Molecular Probes) was added. Fluorescent images were acquired using a fluorescence microscope (Eclipse TE2000-E, Nikon) and recorded using an EMCCD camera (iXon, Andor). Filter sets of 465–495 nm excitation, 515–555 nm emission and 530–550 nm excitation, >575 nm emission were used to measure fluorescence intensity from GFP and FM4–64, respectively. The area of cells was calculated by summing the number of fluorescent pixels using ImageJ software (http://rsbweb.nih.gov/ij/). The relative cell size was calculated by measuring at least 45 cells for each condition.

### Microarrays and expression data normalization

Three biological replicates were performed for each condition, resulting in a total of 90 arrays in this study (Supplementary Figure S8). Total RNA was prepared and microarray analysis was performed using an Affymetrix GeneChip^®^ system as described elsewhere (29,40). A high-density DNA microarray was utilized with the Affymetrix GeneChip system, and data extraction was performed based on the finite hybridization model (41,42) as previously described (29,40). The raw expression data sets were subjected to normalization (43), resulting in a common mean value (logarithmic) in all data sets. To avoid potential noise caused by the small values, normalized expression data with values less than −1.5 were removed, resulting in a total of 3398 genes for subsequent analysis. The averaged expression value for each gene over the three biological replicates was used for computational analyses. Both the normalized expression data sets and the raw CEL files were deposited in the NCBI Gene Expression Omnibus database under the GEO Series accession number (http://www.ncbi.nlm.nih.gov/geo/query/acc.cgi?acc=GSE55719).

### Computational analysis

The Bioconductor software package RankProd (44), which is based on the rank product method (45), was employed to identify differential gene expression (DEGs) caused by histidine depletion. Binomial tests were performed to evaluate the significance of the extracted gene groups using free software packages available from the Broad Institute (http://www.broadinstitute.org). All statistical tests and computational analyses, except for the gene set enrichment analysis (GSEA), were performed using R (39). GSEA (46), which was used to identify the gene groups with significant changes, and PCA (47,48), which was used to classify expression patterns according to gene expression level variance, were performed as previously described (29). PCA converts the high-dimensional data set (i.e. 3398 genes representing 3398 dimensions) into low-dimensional space (e.g. three dimensions). *K*-means clustering was performed as previously described (29). The gene expression levels (logarithmic scale) obtained under histidine-rich conditions were subtracted from those acquired under histidine-depleted conditions. Consequently, two transcriptional change values (−His (2 h) and −His) and a base value of zero (+His) were acquired for each gene in each strain. −His (10 min) data were eliminated because the growth rate contained substantial noise caused by the short time period being measured. *K*-means clustering analysis was performed on this data set, which comprised 22 × 3398 values. PCA was performed on three transcriptional change values (−His (10 min), −His (2 h) and −His), which also comprised 22 × 3398 values.

### Annotation of gene function and regulation

The entire data set of gene names and categories was obtained from GenoBase, Japan (http://ecoli.aist-nara.ac.jp/gb6/Download.html). Transcriptional network information was obtained from RegulonDB v8.0 (49) (http://regulondb.ccg.unam.mx). The transcriptional networks comprising more than 15 regulated genes controlled by a regulator were used in the analysis. MultiFun annotation was performed according to the GenProtEC database (50) (http://genprotec.mbl.edu). The MultiFun classification was applied to annotations comprising >15 genes. The gene categories were in accordance with a previous report (51). GO term annotations for *E. coli* strain K-12 were obtained from the Gene Ontology database (52,53) (http://www.geneontology.org). GO terms (biological processes) that comprised fewer than 15 or greater than 1000 genes were excluded from the analysis.

## RESULTS

### Success and failure in stochastic adaptation

An assortment of rewired *E. coli* strains, i.e. an *OSU12* series (Figure 1A), was employed to evaluate whether cells with rewired genetic structures were able to survive starvation (i.e. the generality of adaptation to disturbed regulation in response to histidine depletion, which was previously demonstrated using a single strain (10)). Each strain carried a gene from the native *His* operon rewired to a monostable synthetic gene circuit (Figure 1A) at another chromosomal location, including a previously reported strain (10). Therefore, these structural genes (*hisG, hisD, hisC, hisB, hisA, hisF* and *hisI*), which are essential for histidine biosynthesis (Figure 1A, I), were no longer subject to their native regulation by the histidine-sensing *His* operon (34–36) but were controlled by the foreign P_tet_ promoter in response to the chemical inducer doxycycline (Figure 1A, II). The expression of the rewired genes was reported by the coexpression of green fluorescent protein (GFP). The native strain (i.e. *OSU11*, Figure 1A, III), which carried the native *His* operon, was utilized as a control, as previously reported (10,37).

Both success and failure were observed among a total of eight strains under histidine starvation. The growth rates of the rewired strains were comparable to the growth rate of the native strain under histidine-rich conditions (Figure 1B, filled), verifying that the genetic reconstruction and the diversity of the rewired genes slightly disturbed cell growth under histidine-rich conditions. However, significant growth decreases, which were accompanied by gene-specific effects, were observed under histidine-depleted conditions (Figure 1B, open). The strains with rewired *hisB, hisC* or *hisF* exhibited relatively high growth fitness that was similar to that of the native strain, indicating that these rewired strains successfully achieved adaptive states that were comparable to the native regulation. Conversely, cell growth was undetectable in the *hisD*- or *hisG*-rewired strains, providing the first cases of failure in adaptation to gene rewiring. Additionally, largely suppressed growth was observed in the *hisA*- or *hisI*-rewired strains, although these strains were sustainable. Such diverse cell fates were commonly observed in the presence of 50–200 nM doxycycline, which determined the basal expression levels of the rewired genes (Supplementary Figure S2). These results provided the first experimental evidence of differentiated cell fates mediated by the stochastic switching of rewired genes under identical conditions.

### Cell fate decision was attributed to fluctuation in rewired genes

**Figure 2.**
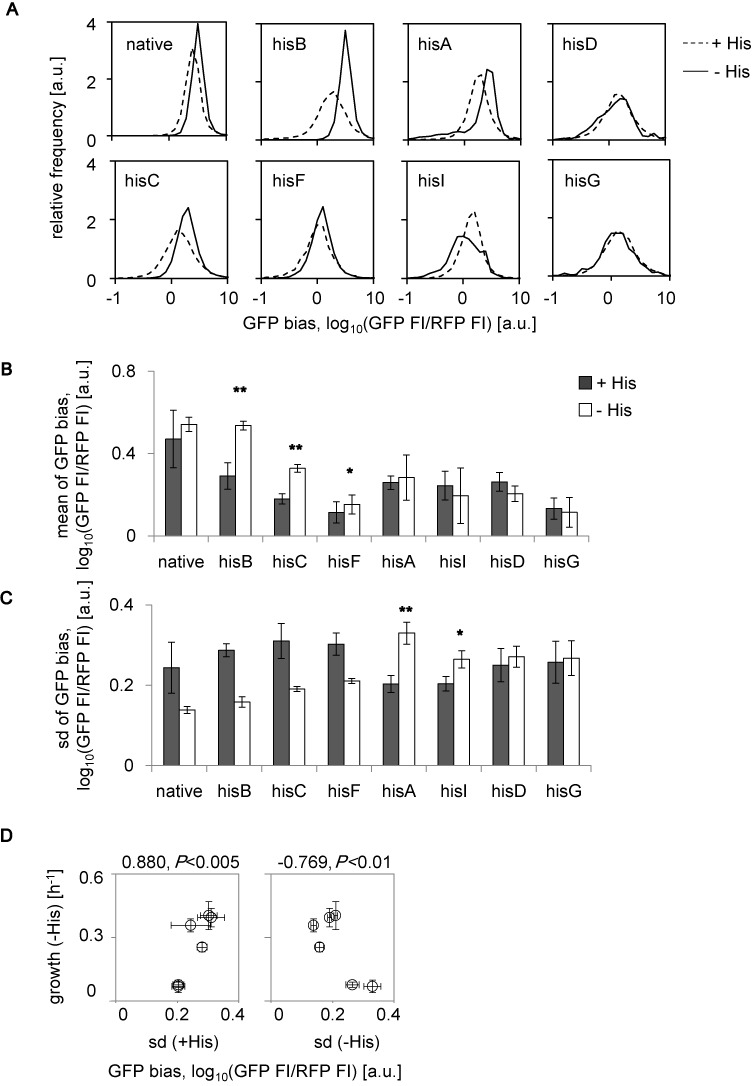
Expression of rewired genes as GFP bias. (**A**) Distributions of GFP bias in the presence and absence of histidine. Steady distributions of the relative cellular GFP bias in the presence (dashed lines) or absence (solid lines) of histidine are shown. The strains are indicated by the names of the rewired genes. (**B**) Average protein abundance. The mean values of the GFP bias in the presence (filled) and absence (open) of histidine are shown. Asterisks indicate significant increases (*P < 0.05 and **P < 0.005). **C.** Cell-to-cell variation in GFP bias. The standard deviation of the GFP bias in the presence (filled) and absence (open) of histidine are shown. Asterisks indicate significant increase as described in (**B**). (**D**) The relationship between growth and variation. The growth rates (Figure 1B, open) are plotted against the standard deviations (shown in **C**) for the data from the native, *hisB, hisC, hisF, hisA* and *hisI* strains. The strains are indicated by the names of the rewired genes. All data sets are on a logarithmic scale. The standard errors of every four independent tests are indicated.

The fluctuating expression of rewired genes was clearly demonstrated in the population distributions of GFP bias, which represents the specific expression of the rewired gene (GFP FI/RFP FI, where RFP was constitutively induced; Figure 2A, Supplementary Figure S3). In the good survivors (*hisB, hisC* and *hisF*), the GFP bias exhibited a strong preference for induced levels in response to histidine depletion, with high statistical significance (Figure 2B, asterisks, Supplementary Figure S3), regardless of the primary mean expression levels (Figure 2B, black). In contrast, such changes were undetected in the poor survivors (*hisA* and *hisI*) or in the failures (*hisD* and *hisG*), although their primary mean expression levels were relatively higher than that in the good survivors of *hisC* and *hisF*.

Notably, the variations in expression within the population were largely related to the final fate, and these results were consistent with the properties of stochastic adaptation. The good survivors exhibited a relatively high degree of cell-to-cell variation within the initial populations compared to the other groups (Figure 2C, filled). The poor survivors exhibited a relatively small initial variation, which tended to increase the variation for adaptation (Figure 2C, asterisks). A positive correlation was found between the initial variation in expression (i.e. standard deviation) and the final adaptiveness (i.e. growth rate) among the six survivors (Figure 2D, left). In addition, a negative correlation between variation and adaptation in histidine depleted conditions (Figure 2D, right) was also found. The selection of adaptive cells may have occurred in the populations exhibiting a large initial variation, such that the variation finally became small in the good survivors. Conversely, an increase in variation most likely occurred in the populations of poor survivors to help breed adaptive cells by chance.

### Changes in cell size compensated for stochastic adaptation

**Figure 3.**
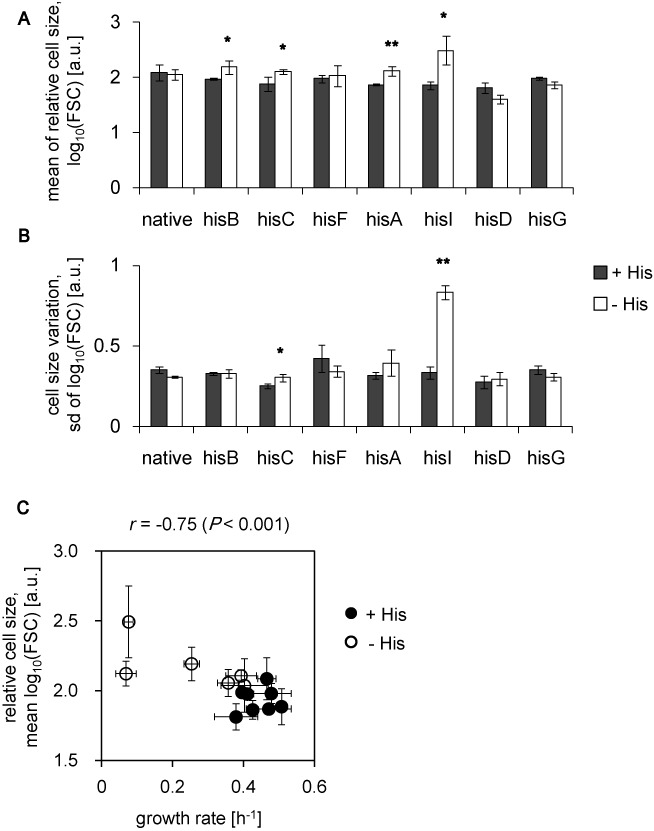
Relative cell size. (**A**) Average cell size. The mean values of the relative cell size, which is represented by the FSC value, in the presence (filled) and absence (open) of histidine are shown. (**B**) Variation in cell size. The standard deviation of the FSC values in the presence (filled) and absence (open) of histidine are shown. The standard errors of every four independent tests are indicated. Asterisks indicate significant increases (*P < 0.05 and **P < 0.001). (**C**) The relationship between cell size and growth in the presence and absence of histidine. Growth rates (Figure 1B) are plotted against mean cell sizes, FCM (Figure 3A). Cells growing in the presence and absence of histidine are indicated as filled and open circles, respectively. The correlation coefficients and corresponding *p* values are indicated. Standard errors are indicated as error bars.

In addition to the fluctuating gene expression (Figure 2), the cell size, as a typical indicator of adaptivity, was also observed. Cell size influences the fluctuated abundance of gene product, which is known as the system dilution effect (54). Therefore, the means and variations of cell sizes within populations were evaluated. Cell size distributions demonstrated the fluctuation of cell size (Supplementary Figure S4), as well as what found in gene expression (Figure 2A, Supplementary Figure S3). The survivors but not the failures tended to increase their cell size in response to histidine depletion (Figure 3A). The increased cell size, specifically in cell length, was quantitatively verified by microscopic observation (Supplementary Figure S5). This filamentation strategy, which was commonly observed in wild-type cells as a stress response (55–57), was assumed to compensate the insufficient fluctuation in gene expression and to facilitate future propagation or generate fluctuations in abundance of gene products by system dilution effect. The strains with large variations in cell size, such as the poor-surviving *hisI*-rewired strain (Figure 3B), may still be in the process of randomly searching for better adaptations.

The slightly increased cell size detected in most strains was correlated with a slightly decreased growth rate under histidine-depleted conditions could be seen (Figure 3C, Supplementary Figure S5C). We reasoned that most cells in the good survivor populations had completely adapted to the stress, such that their cell sizes were slightly increased with only a small variation. Conversely, both adaptive and maladaptive cells were present in the poor survivor populations, and thus the average cell size was large and exhibited a large variation (Figure 3B). The phenotypic plasticity at the morphological level might compensate for the insufficient fluctuations in rewired gene expression, particularly in the poor survivors. The results suggested that the mean expression of the rewired gene was not the only determining factor for adaptation, and the variation of expression within populations and/or the changes in cell size facilitated random searching for an adaptive state.

### Successful cell fate required the cooperative expression of the His operon

**Figure 4.**
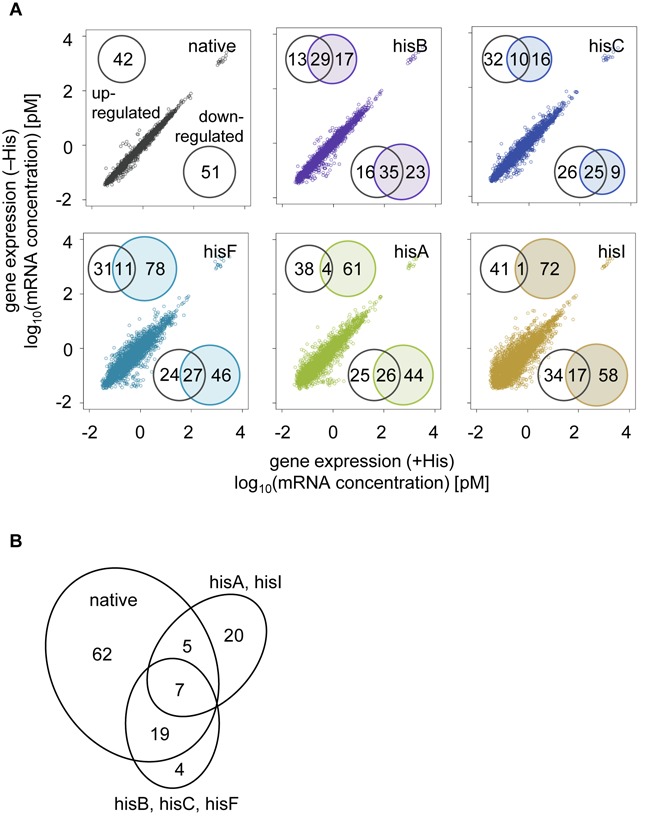
Genome-wide expression. (**A**) Dot plots of gene expression in the presence and absence of histidine. The numbers of differentially expressed genes (DEGs, FDR < 0.05), which are either up- or downregulated, are indicated (inlet). Venn diagrams represent the number of DEGs in the rewired strains that overlap with the native strain. (**B**) The number of overlapping differentially expressed genes. The DEGs that overlap among the strains are shown.

The expression of all rewired genes showed fluctuations to a certain extent (Figures 2 and 3), but the final cell fates (i.e. growth fitness) of these rewired strains in histidine-depleted conditions were dissimilar (Figure 1B). The presence of cooperative gene regulation was further investigated using transcriptome analyses. Six survivors of the same growing conditions that were used for FCM analysis were examined. The expression patterns between histidine-rich and histidine-depleted conditions largely differentiated in the rewired strains compared with the native strain (Figure 4A, dot plots). Several differentially expressed genes (DEGs, determined by the rank product method) were identified in the rewired strains; however, only a few overlapped with those in the native strain especially in poor survivors (Figure 4A, Venn diagrams). Among all DEGs, only seven genes were shared by the survivors (Figure 4B). In addition, gene set enrichment analysis (GSEA), which identified the gene groups and/or networks of significant transcriptional changes in response to histidine depletion, presented large variability (Supplementary Figure S7). These results reflected random patterns of expression at the individual gene or group level in the rewired strains, supported by the previously reports (11,25).

**Figure 5.**
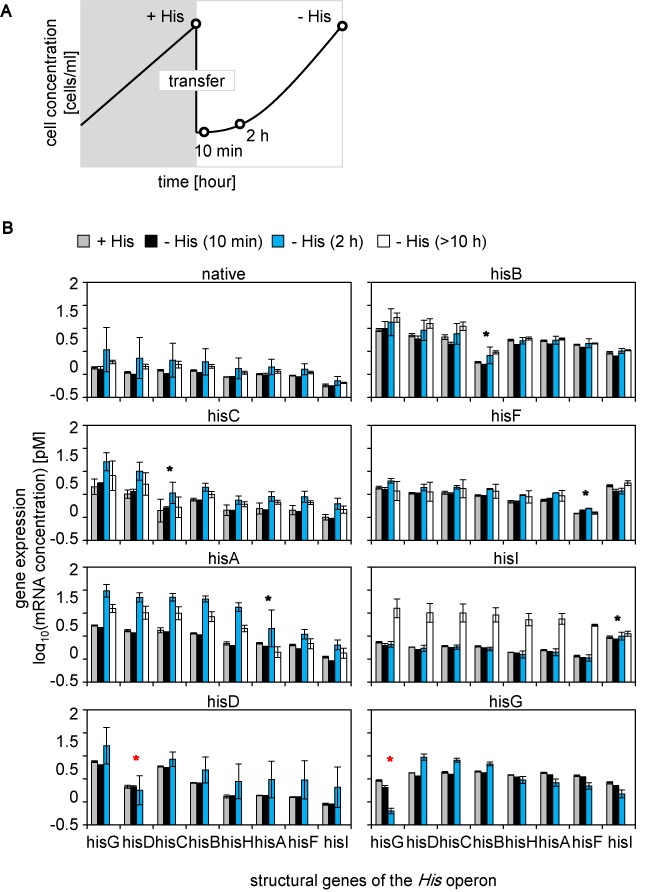
Expression patterns of *His* operon. (**A**) Schematic drawing of sampling points. Open circles indicate the time points of measurements and the samples collected for microarray, +His, −His (10 min), −His (2 h) and –His (>10 h). **B.** The expression levels of the structural genes of the *His* operon. The expression levels initially (+His, gray) and at 10 min (black), 2 h (blue), and >10 h (open) after histidine depletion transfer are shown. Asterisks indicate the rewired genes under foreign control that were isolated from the *His* operon. Standard errors of three biological replicates are indicated.

Additional analyses of the transcriptome changes in the early responsive phase were performed (Figure 5A) because of this failure to obtain common regulatory features in the survivors growing in the exponential phase (Figure 4). Overall, the induced expression of both the rewired gene and the other structural genes of the *His* operon was detected in the survivors in the absence of histidine and was comparable to the expression by the native strain (Figure 5B, Supplementary Figure S9). Such cooperated upregulation of all components in the *His* operon might be necessary for sufficient biosynthesis of histidine. In particular, the structural genes of the *His* operon were highly upregulated 2 h after histidine depletion in most strains (Figure 5B, blue), following a 10-min delay for significant changes (Figure 5B, black). Induced expression of the rewired genes in the corresponding rewired strains, either in early responsive (2 h) or later exponential phases (>10 h), were crucial for the successful cell fate (Figure 5B, black asterisks), which was consistent to FCM analyses (Figure 2). Strains that failed to maintain high expression levels at 2 h (Figure 5B, red asterisks) finally succumbed to maladaptation. Success appeared to largely depend on the original location of the rewired gene within the *His* operon. The strains carrying genes (*hisG* and *hisD*) that were rewired from loci close to the highly conserved ‘attenuator’ (35–36,58) failed to maintain the induced expression of these essential rewired genes. The chromosomal locations of genes may limit cellular plasticity, which is consistent with previous findings on the relationship between chromosome structure and plasticity in gene expression (59).

### Cooperative global transcriptional reorganization guided a success path

**Figure 6.**
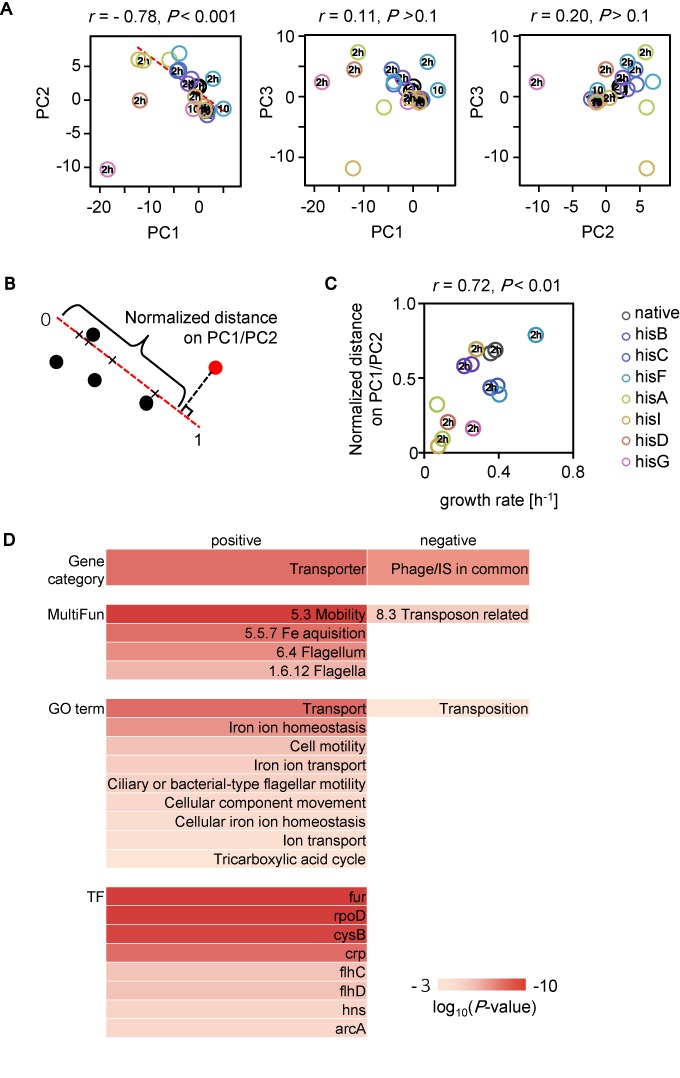
PCA analysis. (**A**) Correlations between each two PCs in PC1, PC2 and PC3 with corresponding *P* values are shown. Transcriptional patterns are described by principal component analysis. The principal component scores PC1, PC2 and PC3 represent 37, 17 and 15% of the total variance, respectively. The ‘10’ circles, ‘2 h’ and open circles represent the states of 10 min, 2 h and >10 h after histidine depletion, respectively. Color variations representing the different strains are indicated. (**B**) Schematic drawing of the calculation of normalized distance. For example, the distance of a certain condition (red filled circle) on the PC1/PC2 correlation line is shown as a basket. (**C**) The relationship between growth and the distance on PC1/PC2. The correlation coefficient and the *P* value are indicated. (**D**) Enriched gene function and regulation. The top 5% of genes positively and negatively (169 genes each) loading on the PC1/PC2 correlation line were used for the enrichment analysis. Gene functions are designated using gene category, MultiFun, and GO terms. Gene regulations are based on transcriptional networks, which are indicated by the names of the transcription factors (TF). Color bars indicate the significance as log-scaled *P* values obtained using binomial tests with Bonferroni corrections (*P* < 0.001).

Principal component analysis (PCA) using all data sets was performed to obtain a conceptual abstract of global cooperation for success in stochastic adaptation. High-dimensional transcriptome reorganizations were primarily restricted within a narrow space and composed of any two PCs of the three main PCs (Figure 6A). In particular, a highly significant correlation was found between the two primary PCs (PC1 and PC2, except for failures), which accounted for ∼54% of the total changes (Figure 6A, left). This negative correlation illustrated a trade-off in gene expression for a homeostatic transcriptome, and a single-directional trajectory allowed for survival (Figure 6A, red dashed line). The large varied distribution of transcriptome changes at 2 h (Figure 6A, 2 h circles) reflected the manner of stochastic adaptation. However, survivors occurred along this trajectory, and the failures dropped off.

Moreover, normalized distances on this PC1/PC2-correlated trajectory showed that growth rates correlated to adaptiveness. The distance from the vertical projection of the circles on the trajectory to a defined initial point on the trajectory was calculated and normalized within one unit (Figure 6B). Data sets of the 10 min bins were excluded because the growth rates that were estimated during this 10 min were unreliable. A positive correlation with high significance was observed between normalized distances and growth rates (Figure 6C), which indicated that transcriptome reorganizations cooperated with the rewired genes to direct successful adaptation. Growth-correlated genes as determined by *K*-means clustering clearly demonstrated that the genes positively and negatively loaded on the PC1/PC2 trajectory mostly comprised clusters that positively and negatively correlated to the growth rates, respectively (Supplementary Figure S8A and B), which strongly supports the finding of the PC1/PC2 trajectory as a success path.

Gene enrichment analysis identified gene functions related to mobility/transport and transposition in the positive and negative loadings, respectively (Figure 6D, Gene category, MultiFun, GO term). *K*-means clustering analysis confirmed that the enriched gene functions concentrated in nonessential processes (Supplementary Figure S8C). These results were quite different from studies on regular adaptation by native regulations, which often reported that central biological functions, such as translation, were enriched in the growth-correlated gene clusters (27,29). In addition, the enriched transcriptional networks in the positive load (Figure 6D, TF, positive) partially overlapped with the networks that were identified in the native response to general stresses (29,40) (e.g. *rpoD*). However, the enrichment of gene regulations failed to identify any regulatory networks with significant changes in the negative loading (Figure 6D, TF, negative), which is consistent with the enriched gene functions. These results indicate that the stochastic adaptation of these rewired strains was also attributable to cooperative reorganization of the expression of those nonessential genes (e.g. phage/IS in common), which is quite different from the well-known stress response mechanisms that are tightly regulated by the corresponding genes (e.g. *rpoS* for general starvation stress). This finding raised an intriguing question whether it was the cooperative transcriptional changes in the nonessential genes that potentially assisted the rewired genes of stochastic switching to stay at the proper expression level. In summary, the single-dimension PC1/PC2 trajectory that was highly abstracted from the multidimensional transcriptome reorganization guided success in stochastic adaptation and the contribution of the cooperative expression of both basal-related and nonessential genes. The population analysis based on the reporter gene *gfp* (Figure 2) and the transcriptome analysis evaluating the genome-wide expression patterns (Figure 6) drew comparable conclusions, in which successful cell fates that are mediated by directional changes in gene expression can be achieved in the absence of specific regulation.

## DISCUSSION

The present study systematically investigated the mechanism of stochastic adaptation that is often proposed to occur *via* successful fluctuations that correspond to the rewired genes. Previous studies have demonstrated that cells possess the ability to cope with the challenge presented by a particular gene being rewired (9–11). That is, cells were induced to search for the proper states independent of the specific regulatory mechanism by the stochastic switching of a certain gene (i.e. the rewired genes) essential for cell growth (10,12–13,20). In this study, the genes originally controlled by the well-known *His* operon were systematically subjected to rewiring. By considering growth recovery (Figure 1B), three different cell fates were identified: highly adaptive (*hisB, hisC* and *hisF*), poorly adaptive (*hisA* and *hisI*), and maladaptive (*hisG* and *hisD*). This survey provides the first experimental evidence regarding the probability (five of seven strains) that a stochastic strategy will succeed under a certain defined condition (i.e. histidine depletion).

**Figure 7.**
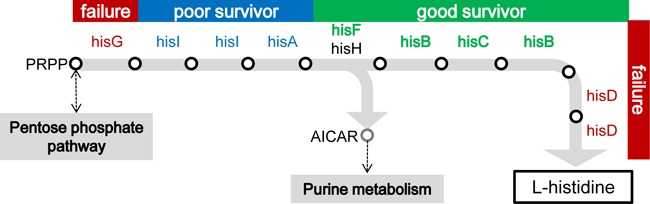
Histidine biosynthetic pathway. A flow chart for histidine biosynthesis was simply constructed according to the KEGG database (61,62). The genes that were subjected to rewiring are highlighted in color. Three types of cell fates are categorized; green (good survivors), blue (poor survivors) and red (failures). The empty circles and shaded arrows represent the intermediate chemicals (metabolic products) and the corresponding enzymatic reactions (metabolic flux), respectively. Except for PRPP (5-phosphoribosyl 1-diphosphate) and AICAR (aminoimidazole carboxamide ribonucleotide), the names of the intermediate chemicals are omitted.

It is intriguing that the strains whose rewired genes were regulated in the same way and were involved in an identical pathway had the different cell fates. First, the success of stochastic adaptation likely depends on the order of either regulation or metabolism. Because *hisG* and *hisD* are the first genes expressed from the *His* operon (Figure 1A), their expression might require highly precise regulation in contrast to other structural genes, which might be loosely controlled under native regulation. In addition to this locus priority in the *His* operon, the function of genes that are involved in the metabolic pathway may play a role. The initial and final steps of histidine biosynthesis are controlled by *hisG* and *hisD*, respectively (Figure 7, red). If the order of gene expression and the order in which a protein plays a role indicate the accuracy of the genetic and metabolic controls (60), then *hisD* and *hisG* must respond in a highly efficient or rapid manner. Transcriptome reorganizations of these two rewired strains may fail to reach the PC1/PC2 trajectory (Figure 6) within the time limit for growth recovery, because random searching for proper expression patterns was time-consuming. Second, the surrounding metabolic pathways (61,62) may influence the adaptivity. The strains carrying the rewired genes participating in the reactions upstream of the bifurcation for purine metabolism (61,62) were poorly adapted (Figure 7, blue), whereas those located downstream were highly adapted (Figure 7, green), indicating that the key metabolic pathway may contribute to the stochastic switching-mediated fate decision and supporting the conclusion that a global cooperative reorganization of gene expression is essential for the success of stochastic adaptation. Third, the *hisF* strain showed a less significant change in GFP bias (Figure 2) and maintained its cell size (Figure 3), compared with the other two good survivors. We assume that the dosage of *hisF* gene product that was required for cell growth might be lower than that for other strains, because this strain showed greater growth fitness than the other rewired strains under all of the induced expression conditions (Supplementary Figure S2). The dose-to-fitness relationship of *hisF* might be different from other genes, most likely because this gene works together with *hisH* (63) in histidine biosynthesis. Because the partner gene *hisH* was maintained under native regulation, the rewiring of *hisF* might be less lethal than the rewiring of other genes that function alone; therefore, perhaps less change is required.

As a first and intriguing finding, the cooperative transcriptional reorganization (Figures 4–6) that occurred globally within a limited space, in addition to the fluctuated expression of the rewired genes and cell size increase, was important for successful stochastic adaptation (Figure 6C). The poor survivors showed a higher degree of global reorganization than the good survivors (Figures 4 and 6), but they all remained along the PC1/PC2 trajectory. This global coordinative change was assumed to occur partially by chance because it was only the phage- and IS-related genes, but not regulators that were significantly enriched in the negative load of the success path (Figure 6D). This nonessentiality in enriched gene functions agreed well with the fact that large cell-to-cell variations of GFP bias were observed in the survivors (Figure 2).

**Figure 8.**
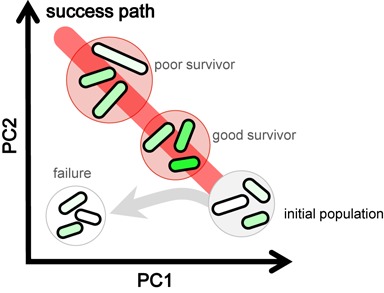
Schematic drawing of coordinated reorganization for adaptation to gene rewiring. A perspective model of cell fate decision is illustrated. Cells are drawn in rod shapes. Fluctuated expression of the rewired gene is represented with the gradations in green, light to dark green, which represents low to high expression. Changes in cell size are indicated as the varied length of the rod shaped drawings. The rewired strain is shown as the population within the circle. The initial and final cell populations are indicated in gray, red or white circles. Detailed information is described in the text.

A perspective model is proposed based on these results. The PC1/PC2 trajectory represents a success path (Figure 8, red bold line) in response to environmental perturbations (e.g. histidine depletion). This path guides the success of stochastic adaptation in gene rewiring in the same direction that is regulated by regular adaptation *via* native regulations. Different cell fates are drawn when the rewired strains carrying damaged regulations encounter unfavorable environments, which is due to the cell-to-cell variation in gene expression and cell size within the initial populations (Figure 8, light gray circle). Cells that luckily switched to the induced expression level of the essential rewired genes (Figure 8, green) in cooperation with synchronized transcriptome reorganization following the success path turned to alive (Figure 8, red circles). Cells of the transcriptome reorganization left this success path (Figure 8, gray arrow) and tended to succumb to death despite stochastic switching of the rewired gene (Figure 8, open circle).

It is unclear whether all 3398 genes had randomly searched for the proper expression levels; nevertheless, the global reorganization occurred directionally along a one-dimensional path. This success path is most likely restricted or shaped by the metabolome, because the cell fates mediated by a stochastic strategy were well categorized from a metabolic perspective (Figure 7). As gene rewiring is thought to be highly likely in natural evolution (1), this one-dimensional path may potentially provide a direction for evolutionary changes at the level of the transcriptome. The distance along this defined path may reveal the evolutionary potential for preexisting gene networks to form novel regulatory connections that are adaptive to unforeseen environments. The observed path was admittedly limited to the context of histidine starvation, but this first successful attempt to search for a general path among 3398 potential dimensions strongly indicates that a general design principle underlies cellular plasticity. This finding illustrates the mechanism of stochastic adaptation and offers novel insights into the systematic design and application of complex gene networks.

## ACCESSION NUMBER

The accession number of the microarray datasets (total 90 arrays) is GSE55719 in the NCBI Gene Expression Omnibus database (http://www.ncbi.nlm.nih.gov/geo/query/acc.cgi?acc=GSE55719).

## SUPPLEMENTARY DATA

Supplementary Data are available at NAR Online.

SUPPLEMENTARY DATA

## References

[1] Carroll S.B. (2005). Evolution at two levels: on genes and form. PLoS Biol..

[2] Davison E. (2001). Genomic Regulatory Systems, Development and Evolution.

[3] Wilkins A.S. (2002). The Evolution of Developmental Pathways.

[4] Li H., Johnson A.D. (2010). Evolution of transcription networks–lessons from yeasts. Curr. Biol..

[5] Habib N., Wapinski I., Margalit H., Regev A., Friedman N. (2012). A functional selection model explains evolutionary robustness despite plasticity in regulatory networks. Mol. Syst. Biol..

[6] Kim J., Kim I., Han S.K., Bowie J.U., Kim S. (2012). Network rewiring is an important mechanism of gene essentiality change. Sci. Rep..

[7] Puthiyaveetil S., Ibrahim I.M., Allen J.F. (2013). Evolutionary rewiring: a modified prokaryotic gene-regulatory pathway in chloroplasts. Philos. Trans. R. Soc. Lond. B Biol. Sci..

[8] Isalan M., Lemerle C., Michalodimitrakis K., Horn C., Beltrao P., Raineri E., Garriga-Canut M., Serrano L. (2008). Evolvability and hierarchy in rewired bacterial gene networks. Nature.

[9] Stolovicki E., Dror T., Brenner N., Braun E. (2006). Synthetic gene recruitment reveals adaptive reprogramming of gene regulation in yeast. Genetics.

[10] Tsuru S., Yasuda N., Murakami Y., Ushioda J., Kashiwagi A., Suzuki S., Mori K., Ying B.W., Yomo T. (2011). Adaptation by stochastic switching of a monostable genetic circuit in Escherichia coli. Mol. Syst. Biol..

[11] Katzir Y., Stolovicki E., Stern S., Braun E. (2012). Cellular plasticity enables adaptation to unforeseen cell-cycle rewiring challenges. PLoS One.

[12] Kashiwagi A., Urabe I., Kaneko K., Yomo T. (2006). Adaptive response of a gene network to environmental changes by fitness-induced attractor selection. PLoS One.

[13] Acar M., Mettetal J.T., van Oudenaarden A. (2008). Stochastic switching as a survival strategy in fluctuating environments. Nat. Genet..

[14] Shimizu Y., Tsuru S., Ito Y., Ying B.W., Yomo T. (2011). Stochastic switching induced adaptation in a starved Escherichia coli population. PLoS One.

[15] Elowitz M.B., Levine A.J., Siggia E.D., Swain P.S. (2002). Stochastic gene expression in a single cell. Science.

[16] Blake W.J., Balazsi G., Kohanski M.A., Isaacs F.J., Murphy K.F., Kuang Y., Cantor C.R., Walt D.R., Collins J.J. (2006). Phenotypic consequences of promoter-mediated transcriptional noise. Mol. Cell.

[17] Bar-Even A., Paulsson J., Maheshri N., Carmi M., O'Shea E., Pilpel Y., Barkai N. (2006). Noise in protein expression scales with natural protein abundance. Nat. Genet..

[18] Newman J.R., Ghaemmaghami S., Ihmels J., Breslow D.K., Noble M., DeRisi J.L., Weissman J.S. (2006). Single-cell proteomic analysis of S. cerevisiae reveals the architecture of biological noise. Nature.

[19] Taniguchi Y., Choi P.J., Li G.W., Chen H., Babu M., Hearn J., Emili A., Xie X.S. (2010). Quantifying E. coli proteome and transcriptome with single-molecule sensitivity in single cells. Science.

[20] Kussell E., Leibler S. (2005). Phenotypic diversity, population growth, and information in fluctuating environments. Science.

[21] Eldar A., Elowitz M.B. (2010). Functional roles for noise in genetic circuits. Nature.

[22] McAdams H.H., Arkin A. (1997). Stochastic mechanisms in gene expression. Proc. Natl. Acad. Sci. U.S.A..

[23] Avery S.V. (2006). Microbial cell individuality and the underlying sources of heterogeneity. Nat. Rev. Microbiol..

[24] Perkins T.J., Swain P.S. (2009). Strategies for cellular decision-making. Mol. Syst. Biol..

[25] Stern S., Dror T., Stolovicki E., Brenner N., Braun E. (2007). Genome-wide transcriptional plasticity underlies cellular adaptation to novel challenge. Mol. Syst. Biol..

[26] Gourse R.L., Gaal T., Bartlett M.S., Appleman J.A., Ross W. (1996). rRNA transcription and growth rate-dependent regulation of ribosome synthesis in Escherichia coli. Annu. Rev. Microbiol..

[27] Brauer M.J., Huttenhower C., Airoldi E.M., Rosenstein R., Matese J.C., Gresham D., Boer V.M., Troyanskaya O.G., Botstein D. (2008). Coordination of growth rate, cell cycle, stress response, and metabolic activity in yeast. Mol. Biol. Cell.

[28] Nahku R., Valgepea K., Lahtvee P.J., Erm S., Abner K., Adamberg K., Vilu R. (2010). Specific growth rate dependent transcriptome profiling of Escherichia coli K12 MG1655 in accelerostat cultures. J. Biotechnol..

[29] Matsumoto Y., Murakami Y., Tsuru S., Ying B.W., Yomo T. (2013). Growth rate-coordinated transcriptome reorganization in bacteria. BMC Genomics.

[30] Acar M., Pando B.F., Arnold F.H., Elowitz M.B., van Oudenaarden A. (2010). A general mechanism for network-dosage compensation in gene circuits. Science.

[31] Schuetz R., Zamboni N., Zampieri M., Heinemann M., Sauer U. (2012). Multidimensional optimality of microbial metabolism. Science.

[32] You C., Okano H., Hui S., Zhang Z., Kim M., Gunderson C.W., Wang Y.P., Lenz P., Yan D., Hwa T. (2013). Coordination of bacterial proteome with metabolism by cyclic AMP signalling. Nature.

[33] Bashor C.J., Horwitz A.A., Peisajovich S.G., Lim W.A. (2010). Rewiring cells: synthetic biology as a tool to interrogate the organizational principles of living systems. Annu. Rev. Biophys..

[34] Lewis J.A., Ames B.N. (1972). Histidine regulation in Salmonella typhimurium. XI. The percentage of transfer RNA His charged in vivo and its relation to the repression of the histidine operon. J. Mol. Biol..

[35] Winkler M.E., Roth D.J., Hartman P.E. (1978). Promoter- and attenuator-related metabolic regulation of the Salmonella typhimurium histidine operon. J. Bacteriol..

[36] Keller E.B., Calvo J.M. (1979). Alternative secondary structures of leader RNAs and the regulation of the trp, phe, his, thr, and leu operons. Proc. Natl. Acad. Sci. U.S.A..

[37] Kashiwagi A., Sakurai T., Tsuru S., Ying B.W., Mori K., Yomo T. (2009). Construction of Escherichia coli gene expression level perturbation collection. Metab. Eng..

[38] Tsuru S., Ichinose J., Kashiwagi A., Ying B.W., Kaneko K., Yomo T. (2009). Noisy cell growth rate leads to fluctuating protein concentration in bacteria. Phys. Biol..

[39] Ihaka R., Gentleman R. (1996). R: A language for data analysis and graphics. J. Comput. Graph. Stat..

[40] Ying B.W., Seno S., Kaneko F., Matsuda H., Yomo T. (2013). Multilevel comparative analysis of the contributions of genome reduction and heat shock to the Escherichia coli transcriptome. BMC Genomics.

[41] Ono N., Suzuki S., Furusawa C., Agata T., Kashiwagi A., Shimizu H., Yomo T. (2008). An improved physico-chemical model of hybridization on high-density oligonucleotide microarrays. Bioinformatics.

[42] Ono N., Suzuki S., Furusawa C., Shimizu H., Yomo T. (2013). Development of a physical model-based algorithm for the detection of single-nucleotide substitutions by using tiling microarrays. PLoS One.

[43] Bolstad B.M., Irizarry R.A., Astrand M., Speed T.P. (2003). A comparison of normalization methods for high density oligonucleotide array data based on variance and bias. Bioinformatics.

[44] Hong F., Breitling R., McEntee C.W., Wittner B.S., Nemhauser J.L., Chory J. (2006). RankProd: a bioconductor package for detecting differentially expressed genes in meta-analysis. Bioinformatics.

[45] Breitling R., Armengaud P., Amtmann A., Herzyk P. (2004). Rank products: a simple, yet powerful, new method to detect differentially regulated genes in replicated microarray experiments. FEBS Lett..

[46] Subramanian A., Tamayo P., Mootha V.K., Mukherjee S., Ebert B.L., Gillette M.A., Paulovich A., Pomeroy S.L., Golub T.R., Lander E.S. (2005). Gene set enrichment analysis: a knowledge-based approach for interpreting genome-wide expression profiles. Proc. Natl. Acad. Sci. U.S.A..

[47] Venables W.N., Ripley B.D. (2002). Modern Applied Statistics with S.

[48] Mardia K.V., Kent J.T., Bibby J.M. (1979). Multivariate Analysis.

[49] Salgado H., Peralta-Gil M., Gama-Castro S., Santos-Zavaleta A., Muniz-Rascado L., Garcia-Sotelo J.S., Weiss V., Solano-Lira H., Martinez-Flores I., Medina-Rivera A. (2012). RegulonDB v8.0: omics data sets, evolutionary conservation, regulatory phrases, cross-validated gold standards and more. Nucleic Acids Res..

[50] Serres M.H., Riley M. (2000). MultiFun, a multifunctional classification scheme for Escherichia coli K-12 gene products. Microb. Comp. Genomics.

[51] Riley M., Abe T., Arnaud M.B., Berlyn M.K., Blattner F.R., Chaudhuri R.R., Glasner J.D., Horiuchi T., Keseler I.M., Kosuge T. (2006). Escherichia coli K-12: a cooperatively developed annotation snapshot–2005. Nucleic Acids Res..

[52] Camon E., Magrane M., Barrell D., Binns D., Fleischmann W., Kersey P., Mulder N., Oinn T., Maslen J., Cox A. (2003). The Gene Ontology Annotation (GOA) project: implementation of GO in SWISS-PROT, TrEMBL, and InterPro. Genome Res..

[53] Dimmer E.C., Huntley R.P., Alam-Faruque Y., Sawford T., O'Donovan C., Martin M.J., Bely B., Browne P., Mun Chan W., Eberhardt R. (2012). The UniProt-GO Annotation database in 2011. Nucleic Acids Res..

[54] Locke J.C.W., Young J.W., Fontes M., Jimenez M.J.H., Elowitz M.B. (2011). Stochastic pulse regulation in bacterial stress response. Science.

[55] Walker G.C. (1984). Mutagenesis and inducible responses to deoxyribonucleic acid damage in Escherichia coli. Microbiol. Rev..

[56] Koga T., Katagiri T., Hori H., Takumi K. (2002). Alkaline adaptation induces cross-protection against some environmental stresses and morphological change in Vibrio parahaemolyticus. Microbiol. Res..

[57] Zaritsky A., Woldringh C.L., Einav M., Alexeeva S. (2006). Use of thymine limitation and thymine starvation to study bacterial physiology and cytology. J. Bacteriol..

[58] Henkin T.M., Yanofsky C. (2002). Regulation by transcription attenuation in bacteria: how RNA provides instructions for transcription termination/antitermination decisions. Bioessays.

[59] Esnault E., Valens M., Espeli O., Boccard F. (2007). Chromosome structuring limits genome plasticity in Escherichia coli. PLoS Genet..

[60] Nishizaki T., Tsuge K., Itaya M., Doi N., Yanagawa H. (2007). Metabolic engineering of carotenoid biosynthesis in Escherichia coli by ordered gene assembly in Bacillus subtilis. Appl. Environ. Microbiol..

[61] Kanehisa M., Goto S. (2000). KEGG: kyoto encyclopedia of genes and genomes. Nucleic Acids Res..

[62] Kanehisa M., Goto S., Sato Y., Kawashima M., Furumichi M., Tanabe M. (2014). Data, information, knowledge and principle: back to metabolism in KEGG. Nucleic Acids Res..

[63] Alifano P., Fani R., Lio P., Lazcano A., Bazzicalupo M., Carlomagno M.S., Bruni C.B. (1996). Histidine biosynthetic pathway and genes: structure, regulation, and evolution. Microbiol. Rev..

