# Supplementary material for: Global coordination in adaptation to gene rewiring
Source: Nucleic Acids Res. 2015 Jan 6;43(2):1304–16. doi: 10.1093/nar/gku1366 (PMC4333410; doi:10.1093/nar/gku1366)
Supplement: SUPPLEMENTARY DATA [file supp_43_2_1304__index.html]

Global coordination in adaptation to gene rewiring — SUPPLEMENTARY DATA 

# Global coordination in adaptation to gene rewiring

## SUPPLEMENTARY DATA

**Files in this Data Supplement:**

- SUPPLEMENTARY DATA
