# Supplementary material for: Global coordination in adaptation to gene rewiring
Source: Nucleic Acids Res. 2015 Jan 6;43(2):1304–16. doi: 10.1093/nar/gku1366 (PMC4333410; doi:10.1093/nar/gku1366)
Supplement: SUPPLEMENTARY DATA [file supp_gku1366_nar-02868-n-2014-File003.docx]

**Global coordination in adaptation to gene rewiring**

Yoshie Murakami *et al.*

**Supplementary Figures S1-S9 p. 2-10**

**Figure S1. Stochastic adaptation of rewired cells. A.** Stochasticity in gene expression provides fluctuation (cell-to cell variation), even in a genetically identical population (dotted line). This fluctuation allows cells that are occasionally born with better state shapes to develop into a new population with higher fitness (red dashed arrow). **B.** Gene rewiring. One of the structural genes rewired from the native regulation (within the *His* operon) for foreign regulation, whose expression is reported by *gfp*.

**Figure S2. Growth profiles under doxycycline induction.** The native and rewired strains were cultured in the absence (-His) or presence (+His) of 1 mM histidine and supplied with different concentrations of doxycycline, varying from 0 to 200 nM. The growth rates were calculated and are shown as a heat map. Color variations from pale yellow to green represent the growth rates from low to high.

**Figure S3. Distributions of GFP bias in the presence and absence of histidine.** Steady distributions of the relative cellular GFP bias in the presence and absence of histidine are indicated by dashed and solid lines, respectively. Biological replicates in addition to those shown in Figure 2A are shown. The strains are indicated by the names of the rewired genes.

**Figure S4. Distributions of relative cell size in the presence and absence of histidine.** Steady distributions of the relative cell size in the presence and absence of histidine are indicated by dashed and solid lines, respectively. Four biological replicates for calculation of the result of Figure 3 are shown. The strains are indicated by the names of the rewired genes.

**Figure S5. Microscopic observation of cell size. A.** Fluorescence images of cells grown in the absence of histidine. Merged images are shown with the green fluorescence of GFP (cytosol) and red fluorescence of FM4-64 (membrane). The scale bars represent 10 μm. **B.** Histograms of the cell size distributions estimated from microscopic observation. The strains are indicated by the names of the rewired genes. **C.** The relationship between cell size and growth in the presence and absence of histidine. Growth rates (Figure 1B) are plotted against mean cell sizes (**B**). Cells growing in the presence and absence of histidine are indicated as filled and open circles, respectively. The correlation coefficients and corresponding *p* values are indicated. Standard errors are indicated as error bars.

**Figure S6. Box plots of gene expression.** Individual results from repeated experiments (in triplicate for each condition) are shown. The expression of 3,398 genes in the eight strains cultured in the presence of histidine, 10 min or 2 h following histidine depletion, and in the absence of histidine are shown. The color variation represents the difference in the strains. The expression levels represent log-scale mRNA concentrations (pM). A total of 90 arrays are displayed.

**Figure S7. Transcriptional reorganization at the gene category and network levels.** Transcriptional changes in response to histidine depletion were evaluated. The results of gene set enrichment analysis, in terms of gene category (**A)** and transcriptional network (**B)** are shown as heat maps. Significance is represented by the color gradation on a logarithmic scale. Vivid yellow and brilliant blue represent high significance for up- and downregulation, respectively. Columns and rows represent strains, which are indicated by the names of the rewired genes, and gene category (**A**) or transcriptional network (**B**), respectively. Transcriptional networks are indicated by the names of the transcription factors.

**Figure S8. *K*-means clustering analysis (*K* = 3). A.** The relationship between the growth rate and gene clusters. Three gene clusters (C1–3), comprising various numbers of genes (n), were defined. According to the correlation coefficients (indicated by *p* values), both positive (C2) and negative (C1) correlations were found between growth rates and changes in gene expression after histidine depletion. The color variation represents the different strains as indicated by the names of the rewired genes. The open circles and “2 h” circles represent the changes in gene expression between the presence of histidine “+” circles (all are zero at the y-axis) and the absence of histidine or 2 h following histidine depletion, respectively. **B.** Gene clusters. The top 5% of genes positively (blue) or negatively (red) loaded on the PC1/PC2-correlated trajectory were classified according to the gene clusters (C1-C3). The numbers of the genes comprising each cluster are shown. **C.** Enriched gene function and regulation. Gene functions are designated by gene category, MultiFun, and GO term. The gene regulations are based on transcriptional networks indicated by the names of the transcription factors (TF). Color bars indicate the significance in log-scaled *p* values obtained using binomial tests with Bonferroni corrections (*p* < 0.001).

**Figure S9 Transcriptional changes of *His* operon.** The expression changes of structural genes of the *His* operon are shown. The expression changes are against initial state levels (+His). Expression changes at 10 min (black filled), 2 h (blue filled), and >10 h (open) after histidine depletion transfer are shown. Asterisks indicate the rewired genes under foreign control that were isolated from the *His* operon. Standard errors of three biological replicates are indicated.
